# Assessment of Qatar’s Health Care Community Call Center Efficacy in Addressing COVID-19 Pandemic Health Care Challenges: Cross-Sectional Study

**DOI:** 10.2196/42753

**Published:** 2024-01-18

**Authors:** Muhammad Atif Waheed, Lolwa Al Mannai, Hanan Khudadad, Jamil Alenbawi, Mariama Aminata Mansaray, Samya Al Abdulla

**Affiliations:** 1 Primary Healthcare Corporation, Qatar Doha Qatar

**Keywords:** COVID-19, COVID Response Service, community call center, virtual consultations

## Abstract

**Background:**

The global COVID-19 pandemic caused by SARS-CoV-2 created many unprecedented challenges for health care organizations worldwide, placing a great deal of strain on the health care systems, especially access to health care services. To address these challenges, Qatar established a centralized digital platform as a community call center, initially offering digital consultations via its hotline (number: 16000) and later expanding to include a COVID-19 vaccination hotline (number: 7077) for mass immunization.

**Objective:**

This study aims to comprehensively examine the community call center’s operations and their significant role during the COVID-19 pandemic.

**Methods:**

Retrospective data were collected from the Health Information and Technology Department of the Primary Health Care Corporation, Qatar, from March 29, 2020, to January 27, 2022. Data analysis for the hotline (number: 16000) focused on telephone and video call volumes, call response rates, abandonment rates, and call classification. In addition, data from the COVID-19 vaccination hotline (number: 7077) were analyzed for call volumes, call response rates, abandonment rates, appointment booking rates, confirmations, rescheduling, and cancellations.

**Results:**

The hotline (number: 16000) received a substantial total of 429,212 calls, with 284,849 (66.37%) calls effectively answered. The average number of calls received per day during the study period was 640.61 (SD 470.53), and the average number of calls answered per day was 425.14 (SD 206.64). Notably, of the total 128,468 consultations, video consultations were conducted for 3810 (2.96%). Among the diverse call categories, diabetes mellitus (6284/84,299, 7.45%), prescriptions and medications (4709/84,299, 5.59%), hypertension (3874/84,299, 4.6%), vitamin D-related issues (3770/84,299, 4.47%), upper respiratory tract infections (2690/84,299, 3.19%), and COVID-19–related inquiries (2590/84,299, 3.07%) were most frequently addressed. For the COVID-19 vaccination hotline (number: 7077), an impressive total of 1,512,354 calls were received, with a 58.27% (n=881,305) call response rate. The average number of calls per day during the study period was 3828.74 (SD 2931.94), and the average number of calls answered per day was 2231.15 (SD 1496.02). Appointment booking accounted for 26.37% (265,721/1,007,596), appointment confirmation accounted for 10.24% (103,136/1,007,596), rescheduling accounted for 7.95% (80,124/1,007,596), and cancellations accounted for 1.6% (16,128/1,007,596) of the calls.

**Conclusions:**

The findings of this research highlight the crucial significance of the community call center hotline (number: 16000) and the COVID-19 vaccination hotline (number: 7077) in effectively addressing the multifaceted challenges posed by the global COVID-19 pandemic. In Qatar, the community call center emerged as an indispensable and accessible centralized resource, facilitating streamlined digital consultations and vaccination appointments. The impressive call response rate highlights its operational efficiency, adeptly managing a diverse range of health-related issues. This study emphasizes the critical role of community call centers in health care emergency response, signaling their potential as invaluable assets for future preparedness and effective mitigation strategies during similar public health crises.

## Introduction

### Background

SARS-CoV-2, an enveloped single-stranded RNA virus with an epicenter in the Hubei province of China, has caused the current COVID-19 pandemic [1]. It initially appeared as an outbreak of an unknown cause of pneumonia and marked the seventh coronavirus outbreak [2]. Following its emergence, it rapidly spread globally, resulting in significantly high morbidity and mortality rates. At the time of writing this paper, on August 18, 2022, there were 589,680,368 confirmed cases of COVID-19 globally, with 6,436,519 deaths and a fully vaccinated population of 4,857,273,828 [3]. In Qatar, 7,355,038 doses of COVID-19 vaccination were administered until the date of writing this paper.

The COVID-19 pandemic poses many challenges to health care systems worldwide and places considerable strain on them. One of the main challenges is to respond to the crisis and simultaneously maintain the provision of essential health care services, which is crucial for a high-quality and resilient health care system. During the COVID-19 pandemic, many essential health care services were disrupted, including cancer screening, tuberculosis screening, HIV testing, outpatient services, and maternal and child health services [4]. The lack of health care workers, diversion of health care staff to COVID-19 management, cancellation of planned treatments, and risk of viral transmission during on-site patient visits have disrupted health care services [5]. It is essential to take necessary steps to curtail the spread of COVID-19 using available resources and the best use of digital technologies [6]. Different countries have responded differently to the COVID-19 crisis. In the United Kingdom, the National Health Services established a COVID Response Service, accessible via 111 phone lines, with the recruitment of 5000 call handlers and 1500 retired clinicians [7]. The Gulf Cooperation Council (GCC) countries implemented various measures in response to the pandemic, including lockdowns of major cities, airline suspensions, school and university closures, restrictions on social gatherings and sporting events, free health care provision, and active screening for COVID-19 [8]. In Saudi Arabia, the Umrah pilgrimage was suspended, and travel restrictions were placed on GCC citizens who had visited COVID-19–affected countries [9]. To protect public health as a national health strategy in Qatar, the Ministry of Public Health has provided digital solutions by providing remote access channels to health care services at the Primary Health Care Corporation (PHCC) and Hamad Medical Corporation in collaboration with the TASMU Smart Program Qatar, Ministry of Transport and Communication, Hukoomi, and Qatar Post, along with notable digital solution providers [10]. A hotline (number: 16000) was set up to provide 24/7 service to patients’ inquiries regarding COVID-19. In addition, the PHCC established an inbound community call center on March 29, 2020, accessible via the hotline (number: 16000), to provide digital virtual primary care consultations as an alternative to face-to-face health center visits. Physicians and nurses working in health centers who were deemed to be at a high risk for COVID-19, such as those with chronic conditions (hypertension, diabetes, ischemic heart disease, chronic kidney disease, pregnancy, immunocompromised state, etc), were given the option of working in the community call center. On January 5, 2021, the community call center added a COVID-19 vaccination hotline (number: 7077) for booking, cancelling, and rescheduling COVID-19 vaccination appointments [11].

The community call center hotline (number: 16000) provides telephone and video consultation services to all registered patients from 28 health centers in Qatar. Individuals can access the call center through hotline (number: 16000) and book appointments for telephone or video consultation with physicians, dentists, and ophthalmologists from 7 AM to 11 PM. Initially, the calls were triaged by the nurses. For patients who are not registered with the health centers, such as visitors and single workers, the triage nurses direct them to the appropriate service. In cases of emergencies requiring immediate medical attention, patients are guided to dial 999 for ambulance services as appropriate. For nonemergency situations, visitors are directed to local health centers, whereas single male workers are referred to worker’s health centers (HC-21), which are operated by the Qatar Red Crescent Society (QRCS). A nurse-led telephone triage service is available from 11 PM to 7 AM, which signposts patients to appropriate services [12]. Although primarily established for the COVID-19 pandemic, it provided all types of consultations, whether urgent or nonurgent. There were 7 physicians and 9 nurse stations. There was 1 ophthalmology station, whereas for the COVID-19 vaccination hotline (number: 7077), there were 22 stations. The staffing level of the community call center varied according to its operational needs during the study period.

Digital consultations offer several advantages for patients, providers, and health care systems [13]. Patients benefit from avoiding waiting in queues, reducing travel burdens [14], convenience, cost efficiency [15], accessibility [16], and high levels of satisfaction in primary care settings [17]. Satisfaction levels are particularly high in digital consultations, which include communicating with physicians, addressing patients’ concerns and queries, developing treatment plans, improving illness comprehension, and offering usefulness and reliability [5]. During pandemics, digital consultations provide an excellent alternative to traditional face-to-face consultations for patients [18]. Providers also experience advantages, including flexible working hours, the ability to work from anywhere; a reduced risk of infection; increased job satisfaction [13,19,20]; and less psychological distress, burnout [13], and sickness, which can be a burden on the organization. From an organizational perspective, digital consultations provide centralized operations with weekly statistics and demand forecasting, eliminating unnecessary patient visits to health centers and resulting in smooth operations and reduced clinic congestion [13]. Moreover, they enable health care services in remote areas and offer an opportunity for service expansion whenever possible. In addition, they contribute to lower CO_2_ emissions and cost savings [20].

Call centers can serve as central hubs to respond to public health emergencies by providing rapid information transfer to health care providers [21]. During the COVID-19 pandemic, telehealth call centers played a significant role in supporting rural community health workers in Uganda, facilitating prompt identification and referral of COVID-19 cases for appropriate care [22]. In South Korea, the telehealth system provided up-to-date information to callers, helping them protect themselves and others from COVID-19 effectively [23]. Similarly, China established psychological support hotlines to offer mental health assistance during the pandemic [24], and in Paris, France, health care workers benefited from psychological support services [25].

### Objective

Despite the importance of call centers during the pandemic, there is limited information available on their operations and outcomes, especially in relation to the COVID-19 pandemic and the vaccination hotline. Existing literature does not include reports from GCC countries detailing the role and impact of community call centers during the pandemic. This study aims to fill this gap by examining call center operations; documenting their contributions; and analyzing call volumes, patterns, response rates, categories of calls, priorities, and problems or diagnoses. By investigating the effectiveness of community call centers in Qatar during the COVID-19 pandemic, this research seeks to provide valuable insights into their performance during public health emergencies.

## Methods

### Study Design

A cross-sectional study design was used to assess the community call center hotline operations and performance during the study period.

### Disease and Study Population

This research mainly evaluated community call center services to handle the COVID-19 pandemic health care challenges, specifically focusing on their utilization and effectiveness. The study population consisted of patients seeking routine and urgent consultations and advice regarding COVID-19 and its vaccination.

### Location

Data were retrospectively collected from the Health Information and Technology Department of the PHCC, Qatar headquarters, where the community call center is located.

### Time Frame

Data for the community call center hotline (number: 16000) were collected from March 29, 2020, to January 27, 2022. Similarly, data for the community call center vaccination hotline (number: 7077) were collected from December 29, 2021, to January 27, 2022. This time frame encompassed critical phases of the COVID-19 pandemic and the subsequent mass vaccination campaign.

### Data Collection and Analysis

This study involved meticulous analysis of 2 separate data sets: one pertaining to the hotline service (number: 16000) and the other concerning the COVID-19 vaccination hotline (number: 7077). The hotline service (number: 16000) data set was analyzed for various essential metrics, including call volume, percentage of calls answered or abandoned, call patterns, categories of calls, and specific health concerns addressed. Similarly, the COVID-19 vaccination hotline (number: 7077) data set was thoroughly scrutinized, considering call volume; call response rates; call patterns; and appointment-related information such as bookings, rescheduling, confirmations, and cancellations.

### Data Cleaning

To ensure utmost data quality and reliability, a robust data cleaning process was meticulously executed. The 3-step approach encompassed vigilant screening of the databases to detect and address any suspicious features, precise diagnosis of faulty data to ensure accurate results, and appropriate treatment of identified discrepancies. Moreover, a separate copy of the original data was meticulously created in a new file to maintain data integrity during the cleaning process.

### Data Reporting

Conforming to the highest standards of scientific reporting, this study adhered to the RECORD statement, an extension of the STROBE (Strengthening the Reporting of Observational Studies in Epidemiology) statement checklist. This ensured accurate and transparent reporting of the secondary data analysis, further enhancing the credibility of the study findings [26].

### Ethical Considerations

This study obtained ethical approval from the PHCC Research Subcommittee, adhering to research ethics and ensuring participant protection (PHCC/DCR/2021/11/068). As the study used existing deidentified data, informed consent was waived in accordance with the approved protocol. Stringent privacy and confidentiality measures were implemented to safeguard the data. The study strictly complied with data protection regulations to maintain the confidentiality of sensitive information. No compensation was provided to human participants, as the study involved secondary analysis of deidentified data and did not involve direct interaction with participants. The research team took utmost care to handle the data responsibly and ethically throughout the study.

## Results

### Community Call Center Hotline (Number: 16000)

Table 1 shows the quarterly performance of the community call center hotline (number: 16000). During the study period, 66.37% (284,849/429,212) of the calls were handled, with the highest being handled in the third quarter (33,395/34,376, 97.15%), followed by the fourth quarter (34,543/35,885, 96.26%) of 2021. The average number of calls per day during the study period was 640.61 (SD 470.53), and the average number of calls answered per day was 425.14 (SD 206.64).

**Figure 1:**
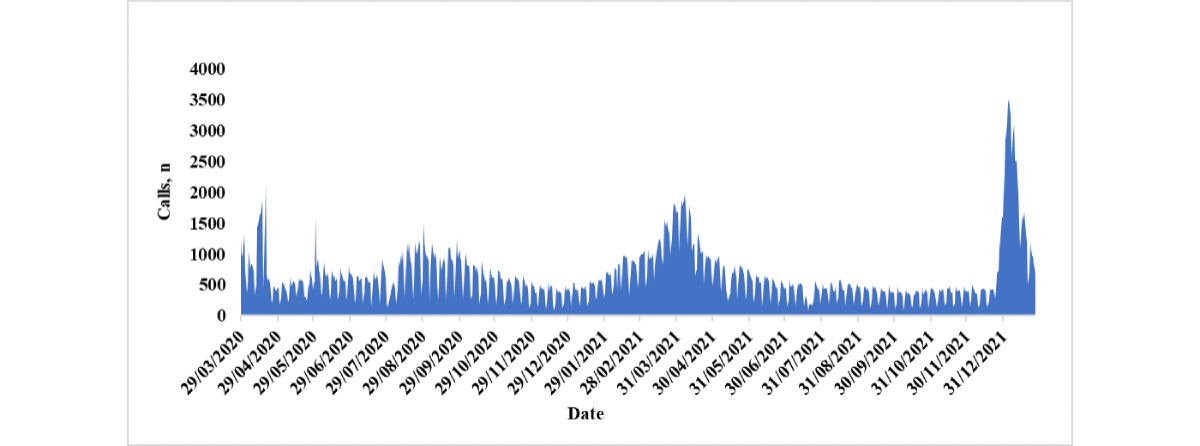
Call volume patterns of hotline (number: 16000).

Figure 1 shows the hotline (number: 16000) call volume patterns during the study period. The peak of calls occurred on April 19, 2020; April 7, 2021; and January 5, 2022.

**Figure 2:**
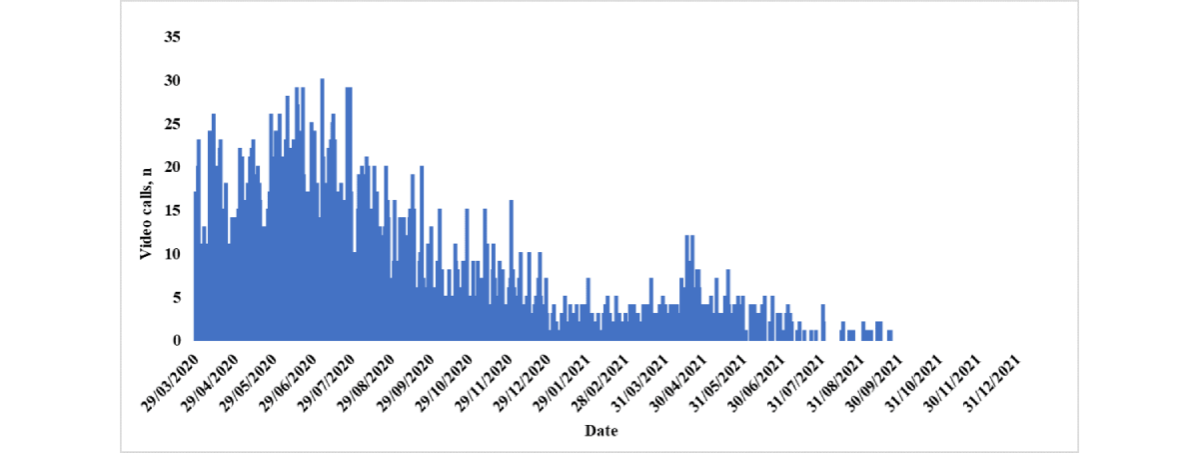
Volume and pattern of video consultations over the hotline (number: 16000).

Figure 2 shows the volume and pattern of the video consultations conducted during the study period. Video consultations accounted for 2.96% (3810/128,468) of the total consultations conducted. The number of video consultations conducted was highest in the beginning of the COVID-19 pandemic reaching to its peak in the first week of July 2020 and then gradually declined.

**Figure 3:**
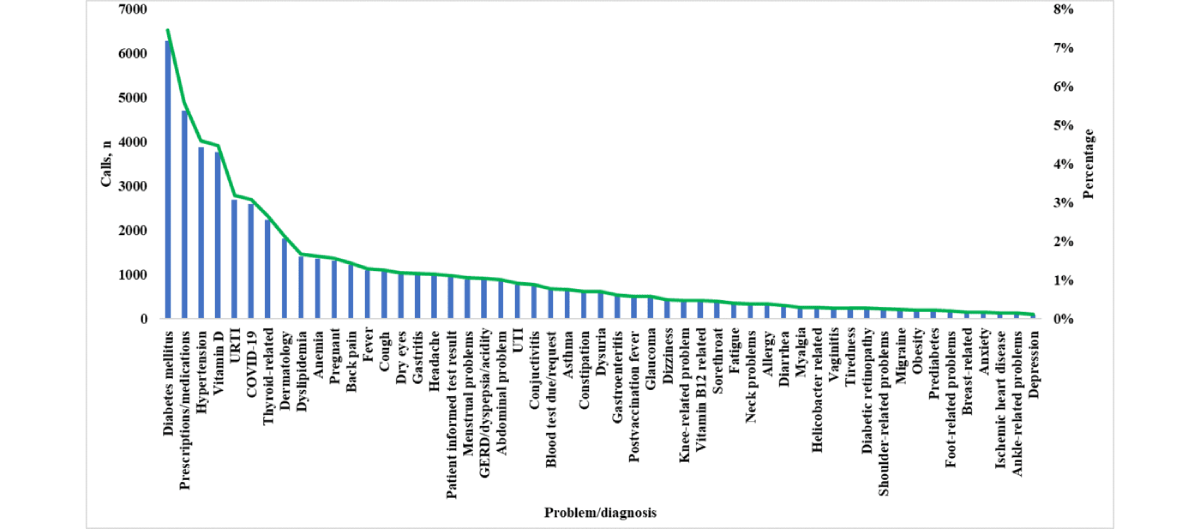
Problem-based summary of consultations over hotline (number: 16000). GERD: gastroesophageal reflux disease; URTI: upper respiratory tract infection; UTI: urinary tract infection.

Figure 3 illustrates problem-based summary of consultations over hotline (number: 16000). The highest number of calls was related to diabetes mellitus (6284/84,299, 7.45%), prescriptions or medications (4709/84,299, 5.59%), hypertension (3874/84,299, 4.6%), vitamin D-related issues (3770/84,299, 4.47%), upper respiratory tract infection (2690/84,299, 3.19%), COVID-19 (2590/84,299, 3.07%), thyroid-related problems (2237/84,299, 2.65%), dermatological problems (1807/84,299, 2.14%), dyslipidemia (1402/84,299, 1.66%), anemia (1358/84,299 , 1.61%), pregnancy (1320/84,299, 1.57%), back pain (1216/84,299 , 1.44%), fever (1089/84,299, 1.29%), cough (1060/84,299, 1.26%), dry eyes (1004/84,299, 1.19%), gastritis (984/84,299, 1.17%), and many other problems or diagnoses.

**Table 1:** Quarterly (Q) performance of community call center hotline (number: 16000).

Time period			Total calls, n (%)		Total calls answered, n (%)		Total calls abandoned, n (%)		Calls/d, mean (SD)		Calls answered/d, mean (SD)		Calls abandoned/d, mean (SD)
**2020**													
	Q1^a^ (n=3146)	3146 (100)		1906 (60.58)		1240 (39.42)		1048.67 (125.43)		635.33 (75.74)		413.33 (200.97)	
	Q2 (n=57,628)	57,628 (100)		37,732 (65.48)		19,896 (34.52)		633.27 (365.44)		414.64 (159.40)		218.64 (223.79)	
	Q3 (n=64,172)	64,172 (100)		37,224 (58.01)		26,948 (41.99)		697.52 (318.96)		404.61 (127.12)		292.91 (218.14)	
	Q4 (n=43,642)	43,642 (100)		27,834 (63.78)		15,808 (36.22)		474.37 (214.21)		302.54 (98.53)		171.83 (137.15)	
**2021**													
	Q1 (n=68,364)	68,364 (100)		35,070 (51.3)		33,294 (48.7)		759.60 (386.49)		389.67 (138.61)		369.93 (283.37)	
	Q2 (n=70,255)	70,255 (100)		49,831 (70.93)		20,424 (29.07)		772.03 (421.53)		547.59 (164.05)		224.44 (304.45)	
	Q3 (n=34,376)	34,376 (100)		33,395 (97.15)		981 (2.85)		373.65 (133.23)		362.99 (128.06)		10.66 (10.31)	
	Q4 (n=35,885)	35,885 (100)		34,543 (96.26)		1342 (3.74)		390.05 (258.57)		375.47 (212.67)		14.59 (59.67)	
Q1 2022^b^ (n=51,744)			51,744 (100)		27,314 (52.79)		24,430 (47.21)		1916.44 (944.09)		1011.63 (220.61)		904.81 (764.96)
Total (n=429,212)			429,212 (100)		284,849 (66.37)		144,363 (33.63)		640.61 (470.53)		425.14 (206.64)		215.46 (311.91)

^a^March 29 until March 31, 2020.

^b^January 1 until January 27, 2022.

### Community Call Center Vaccination Hotline (Number: 7077)

Table 2 shows the quarterly performance of the COVID-19 vaccination hotline (number: 7077). The COVID-19 hotline (number: 7077) handled approximately 58.27% (881,305/1,512,354) of the total calls, whereas 41.73% (631,049/1,512,354) were abandoned during the study period. The average number of calls per day during the study period was 3828.74 (SD 2931.94), whereas the average number of calls answered per day was 2231.15 (SD 1496.02). The highest percentage of calls answered was during the third quarter of 2021 (112,445/118,372, 94.99%), while the lowest number of calls answered was during the first quarter of 2021 (180,205/311,364, 42.12%).

**Figure 4:**
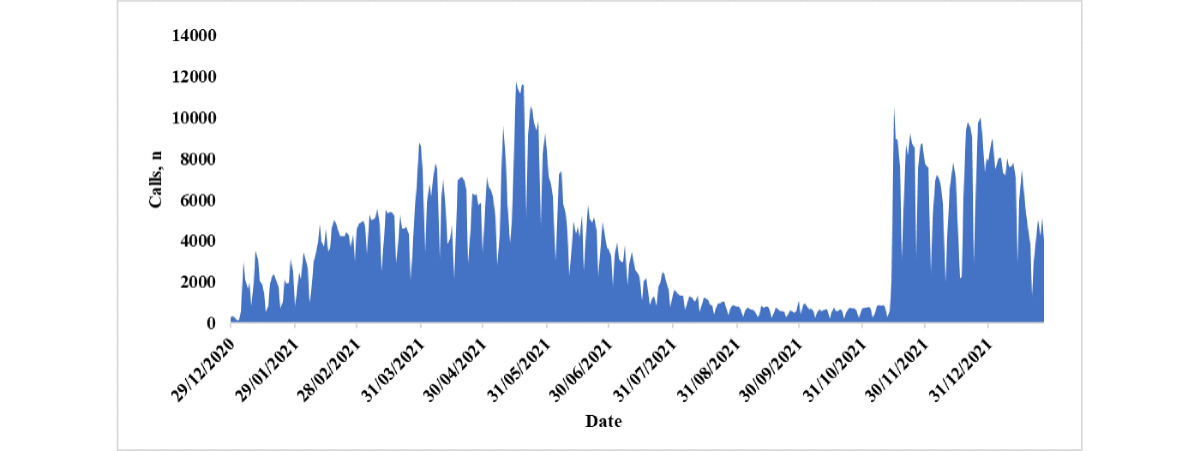
Volume and pattern of calls received by COVID-19 vaccination hotline (number: 7077).

Figure 4 shows the volumes of calls and the pattern received by the COVID-19 vaccination hotline (number: 7077) during the study period. The highest number of calls was received around mid-May and November and the last week of December 2021.

Table 3 displays the quarterly call categories for the COVID-19 vaccination hotline (number: 7077). The highest number of appointments booked occurred in the fourth quarter of 2021 (86,924/1,007,596, 8.63%), followed by that in the second quarter (81,331/1,007,596, 8.07%). Similarly, the highest number of confirmed appointments occurred during the second (51,122/1,007,596, 5.07%) and fourth quarter (16,735/1,007,596, 1.66%) of 2021. The highest number of appointment cancellations occurred in the second (7028/1,007,596, 0.7%) and third quarter (2072/1,007,596, 0.21%) of 2021. Notably, the highest call volume identified for the workers’ health centers (HC-21) operated by QRCS occurred during the second quarter of 2021 (62,208/1,007,596, 6.17%), followed by the fourth quarter of 2021 (43,735/1,007,596, 4.34%). Calls from patients who did not meet the age vaccination criteria, as announced by the Ministry of Public Health of Qatar, were the highest in the second quarter of 2021 (57,081/1,007,596, 5.67%), followed by the third quarter of 2021 (8337/1,007,596, 0.83%).

Table 4 illustrates the distribution of call categories for the COVID-19 vaccination hotline (number: 7077) during the study period. Among the incoming calls, approximately 26.37% (265,721/1,007,596) were associated with appointment bookings, 10.24% (103,136/1,007,596) were for confirming existing appointments, 7.95% (80,124/1,007,596) involved rescheduling appointments, and 1.6% (16,128/1,007,596) were calls to cancel previously scheduled appointments. In addition, 14.8% (149,117/1,007,596) of the calls were identified as related to the workers’ health centers (HC-21) operated by QRCS, while 7.11% (71,600/1,007,596) of the calls received did not meet the age criteria for vaccination.

**Table 2:** Quarterly (Q) performance of COVID-19 vaccination hotline (number: 7077).

Time period		Total calls, n (%)	Calls answered, n (%)	Calls abandoned, n (%)	Calls/d, mean (SD)	Calls answered/d, mean (SD)	Calls abandoned/d, mean (SD)	
Q4 2020^a^ (n=852)		852 (100)	297 (34.86)	555 (65.14)	284 (36.01)	99 (21.52)	185 (57.42)	
**2021**								
	Q1 (n=310,842)	310,842 (100)	131,159 (42.19)	179,683 (57.88)	3453.80 (1735.19)	1457.32 (849.34)	1996.48 (1214.87)	
	Q2 (n=555,336)	555,336 (100)	356,259 (64.15)	199,077 (35.85)	6102.59 (2315.49)	3914.93 (885.59)	2187.66 (1788.70)	
	Q3 (n=118,372)	118,372 (100)	112,445 (94.99)	5927 (5.01)	1286.65 (887.29)	1222.23 (836.57)	64.42 (52.90)	
	Q4 (n=361,461)	361,461 (100)	190,959 (52.83)	170,502 (47.17)	3928.92 (3627.34)	2075.64 (1546.77)	1853.28 (2217.60)	
Q1 2022^b^ (n=165,491)		165,491 (100)	90,186 (54.5)	75,305 (45.5)	6129.30 (2040.44)	3340.22 (650.37)	2789.07 (1792.58)	
Total (n=1,512,354)		1,512,354 (100)	881,305 (58.27)	631,049 (41.73)	3828.74 (2931.94)	2231.15 (1496.02)	1597.59 (1790.57)	

^a^December 29 until December 31, 2020.

^b^January 1 until January 27, 2022.

**Table 3:** Quarterly (Q) call categories of COVID-19 vaccination hotline (number: 7077; n=1,007,596).

Time period		Rescheduled, n (%)	Cancelled, n (%)	Booked, n (%)	Confirmed, n (%)	>1 appointment, n (%)	Worker’s health centers (HC-21), n (%)	Unmet vaccination age criteria, n (%)	Other n (%)
Q4 2020^a^		81 (0.01)	14 (0)	0 (0)	78 (0.01)	13 (0)	0 (0)	0 (0)	68 (0.01)
**2021**									
	Q1	17,164 (1.7)	3514 (0.35)	42,559 (4.22)	13,195 (1.31)	823 (0.08)	0 (0)	0 (0)	62,762 (6.23)
	Q2	24,306 (2.41)	7028 (0.70)	81,331 (8.07)	51,122 (5.07)	1349 (0.13)	62,208 (6.17)	57,081 (5.67)	121,467 (12.06)
	Q3	11,856 (1.18)	2072 (0.21)	20,442 (2.03)	14,737 (1.46)	656 (0.07)	16,006 (1.59)	8337 (0.83)	50,256 (4.99)
	Q4	17,815 (1.77)	1943 (0.19)	86,924 (8.63)	16,735 (1.66)	240 (0.02)	43,735 (4.34)	4132 (0.41)	60,223 (5.98)
Q1 2022^b^		8902 (0.88)	1557 (0.15)	34,465 (3.42)	7269 (0.72)	221 (0.02)	27,168 (2.7)	2050 (0.2)	23,692 (2.35)
Total		80,124 (7.95)	16,128 (1.6)	265,721 (26.37)	103,136 (10.24)	3302 (0.33)	149,117 (14.80)	71,600 (7.11)	318,468 (31.61)

^a^December 29 until December 31, 2020.

^b^January 1 until January 27, 2022.

**Table 4:** Distribution of call categories for COVID-19 vaccination hotline (number: 7077).

Call category	Values (n=1,007,596), n (%)
Booked	265,721 (26.37)
Confirmed	103,136 (10.24)
Rescheduled	80,124 (7.95)
Cancelled	16,128 (1.6)
Worker’s health centers (HC-21)	149,117 (14.8)
Unmet vaccination age criteria	71,600 (7.11)
Other	318,468 (31.61)

## Discussion

### Principal Findings

According to the findings of this study, the community call center hotline (number: 16000) and the COVID-19 vaccination hotline (number: 7077) have played a pivotal role during the ongoing COVID-19 pandemic. These hotlines effectively answered 66.37% (284,849/429,212) of the calls for urgent consultation requests and 58.27% (881,305/1,512,354) of the calls for COVID-19 vaccination appointment inquiries. Notably, the community call center serves as a centralized and versatile resource for citizens and residents of Qatar, offering consultations for a wide range of cases, whether they are emergencies, urgent, or nonurgent, and enabling centralized operations for the mass vaccination program against COVID-19. It has provided a hub during challenging times when health care organizations have witnessed a rapid shift from face-to-face to virtual consultations.

The results of this study showed that after 4 weeks of the first COVID-19 case in Qatar, which was announced on February 29, 2020, the community call center was fully operating. The trajectory of the call volumes received is in line with the peaks of COVID-19 cases, with the first one occurring on May 30, 2020, the second occurring in the middle of April 2021, and the third occurring in the middle of January 2022. As a result, during the COVID-19 pandemic including the lockdown phases, the people of Qatar were able to receive the best medical care. This has also prevented the spread of COVID-19 while providing alternative solutions for patients to access primary health care services [27]. With the community call center, patients have been empowered in terms of flexibility and convenience to use virtual consultations as an alternative to traditional health care services.

The community call center’s diagnostic categories revealed its capacity to handle a diverse array of medical concerns, encompassing inquiries related to COVID-19; vaccination inquiries; acute problems; chronic diseases; medication and repeat prescription requests; requests for laboratory investigations; and various medical conditions related to ear, nose and throat, ophthalmology, dermatology, psychiatry, gynecology, gastrointestinal, neurology, cardiovascular, orthopedics, hematology, pediatrics, and endocrine and metabolic conditions. Interestingly, patients with diabetes mellitus accounted for the highest number of consultations, and virtual consultations have proved effective for managing type 2 diabetes with outcomes comparable with face-to-face consultations, as demonstrated in primary care settings in Australia [28]. Similarly, the management of hypertension via virtual consultations during the COVID-19 pandemic has offered a holistic approach to achieve hypertension control in the United States [29].

It is intriguing to note that video consultations experienced a significant surge in demand during the early phase of the COVID-19 pandemic, especially in the first week of July 2020, reaching a peak of 30 video consultations per day. However, following this peak, the use of video consultation gradually declined over time. Although there is some evidence indicating that video consultations were more sought-after during periods of higher COVID-19 cases, such as in early July 2020 and mid-April 2021, it is essential to highlight that this correlation is not consistently observed throughout the data set. Notably, there were instances of relatively low video consultation counts during times of higher COVID-19 cases, such as in mid-January 2022. The lower use of video consultations can be attributed to various factors, including patients’ preferences influenced by cultural and language barriers, their comfort or inclination toward telephone or face-to-face consultations, privacy and security concerns, and technical limitations. Cultural reasons play a role in shaping patient preferences; some individuals may feel more at ease and familiar with consultation methods such as telephone calls. The content, duration, and quality of video consultations might resemble those of telephone consultations, which could affect patient choices [30], aligning with the findings reported in existing literature. Interestingly, in the United Kingdom, video consultations constitute <1% of consultations in general practice [31]. This preference for other consultation methods, including telephone and face-to-face consultations, might be influenced by physicians’ perceptions that video consultations offer minimal advantages compared with the alternatives.

The COVID-19 vaccination hotline (number: 7077) played a crucial role in Qatar’s mass immunization program, facilitating services for booking, cancellations, and rescheduling initially based on professional group and age criteria. The program was launched on December 21, 2020, introducing the BNT162b2 (Pfizer-BioNTech) messenger RNA vaccine initially, followed by the mRNA-1273 (Moderna) vaccine 3 months later. Initially, priority was given to vaccinating frontline health care workers, individuals with severe or multiple chronic diseases, and those aged ≥70 years. Subsequently, the program was extended gradually by 1 age group at a time, along with selected professional groups, using age as the primary eligibility criterion throughout the rollout [32]. The data from the vaccination hotline revealed noteworthy call volume spikes during mid-May, mid-November, and the last week of December 2021, coinciding with new announcements related to the vaccination program’s expansion. However, the percentage of vaccination hotline’s answers was observed to be lower than that of digital consultation phones. This disparity in the response rates can be attributed to multiple factors. Periods of high demand, such as vaccination eligibility expansions or public announcements about vaccination campaigns, led to a surge in hotline calls, potentially affecting response times due to the increased call volume. Specific eligibility criteria for vaccination appointments also played a role, as some callers may not have met these criteria, resulting in redirection or absence of appointments. Technical issues, waiting times, and call congestion further influenced the hotline’s response rate. Notably, the third quarter of 2021 demonstrated a higher response rate for the vaccination hotline. This could be attributed to a relatively lower volume of calls received during this period, possibly influenced by factors such as expats traveling during summer vacations and the vaccination hotline’s capacity to meet the demand effectively. Understanding these dynamics is crucial for optimizing hotline performance and enhancing vaccination service accessibility. Both vaccines in Qatar have been found to elicit strong protection against COVID-19, prevent hospital admission, and reduce mortality [33]. Due to mass vaccination supported by the community call center and other measures taken by the Ministry of Public Health, Qatar’s mortality remained very low at 0.0016%, whereas globally, it was 1% at the time of writing this paper.

One of the most significant outcomes of the COVID-19 pandemic is that many outpatient appointments can now be managed efficiently via telemedicine without affecting patient care [34]. The COVID-19 pandemic has shown that health care workers can swiftly adjust to the new technologies required to use telemedicine [13]. In a study involving 23 primary care providers and 1692 patients, both providers and patients reported a desire to continue telemedicine visits after the pandemic in primary care settings in the United States [17]. Even before the COVID-19 pandemic, the telehealth business flourished, with a market value of >US $50 billion in 2019 and an anticipated growth rate of more than 9-fold over the next decade [35]. Virtual consultant jobs have also been advertised [36].

The significance of the community call center and vaccination hotline in providing consultations, vaccinations, and health care services during a challenging period demonstrates the potential and value of telehealth solutions in health care systems worldwide. By sharing the successful experiences and best practices of the community call center hotlines, this study can contribute to the enhancement of telehealth services and call centers globally. The lessons learned from operating the hotline can be invaluable for improving crisis response strategies and optimizing health care delivery during public health emergencies. By leveraging digital technologies, health care systems can enhance access to services, improve patient satisfaction, and manage public health emergencies effectively. Integrating call centers and telehealth into routine health care services and emergency response strategies can provide long-term benefits beyond the pandemic.

### Limitations

This study has several limitations. The data presented in this study involved 3 peaks of COVID-19 that affected the overall percentage of calls answered by the hotline (number: 16000). The hotline answered 100% of all calls on several days of the week during the study period. Perhaps, the percentage of calls answered improved to 71.71% at the time of writing this paper. Similarly, for the COVID-19 vaccination hotline (number: 7077), a surge in the volume of calls due to a new announcement of rolling out of the vaccination program affected the overall percentage of the calls answered. COVID-19 vaccination hotline (number: 7077) also answered >97% of the calls on many days of the week during the study period. The percentage of calls handled by the COVID-19 vaccination hotline (number: 7077) improved to 62.17% by the date of writing this paper.

It is important to acknowledge that although this study provides valuable insights into the role of community call center during the COVID-19 pandemic in Qatar, the applicability of these findings to low- and middle-income countries (LMIC) may be subject to limitations. LMIC often face unique challenges, including resource constraints, technological disparities, and varied health care infrastructures. The digital divide prevalent in many LMIC could significantly exacerbate the access-to-technology gap, hindering the establishment and effectiveness of call centers. Moreover, the lack of access to robust health systems or timely responses in LMIC might impact the feasibility and sustainability of implementing similar call center solutions. The success of such initiatives in Qatar, being one of the richest countries globally, may not necessarily translate seamlessly to LMIC because of the nuanced economic, infrastructural, and health care disparities prevalent in those regions. Therefore, although call centers can be a valuable tool, particularly in health care emergencies, their feasibility and effectiveness must be carefully evaluated in the context of LMIC’s unique challenges and constraints.

### Conclusions

This study highlights the significant role played by the community call center hotline (number: 16000) and the COVID-19 vaccination hotline (number: 7077) in effectively managing the challenges caused by the COVID-19 pandemic in Qatar. The community call center responded well to these challenges in Qatar by providing patients with an accessible, centralized resource as an alternative platform for telephone and video consultations and for managing a mass vaccination program. The findings demonstrate the call center’s operational efficiency to handle high call volumes and offer diverse consultations, effectively addressing a wide range of health concerns throughout different waves of the COVID-19 pandemic. Virtual consultations have emerged as a practical solution to empower patients with flexibility and convenience. Although video consultations experienced a temporary surge, their overall use gradually declined during the specified study period, reflecting patients’ preference for other consultation methods. Nevertheless, the community call center remained instrumental in managing various health care aspects, including chronic disease management and COVID-19–related queries. Moreover, the COVID-19 vaccination hotline played a major role in executing a successful mass immunization program. Prioritizing frontline health care workers and susceptible groups initially, the vaccination drive significantly contributed to reducing mortality rates in Qatar. This accomplishment can be attributed to the coordinated efforts of the community call center and other strategic measures implemented by the Ministry of Public Health of Qatar. As the COVID-19 pandemic recedes, it will be essential to adapt the community call center’s capacity and its services to address future health care challenges. Expanding community call center services to encompass specialized health services such as dermatology, counseling, health education, and psychological support could further enhance Qatar’s health care infrastructure. Our study emphasizes the critical significance of telemedicine and digital solutions in crisis response and health care delivery. The insights gained from the community call center in Qatar during the pandemic will provide valuable lessons for improving health care accessibility and emergency response strategies in the future. As health care systems transition to postpandemic norms, leveraging the knowledge and experience gained will foster resilience and efficacy in addressing upcoming challenges, ensuring the well-being of the population.

## Abbreviations

GCC: Gulf Cooperation Council
LMIC: low- and middle-income countries
PHCC: Primary Health Care Corporation
QRCS: Qatar Red Crescent Society
STROBE: Strengthening the Reporting of Observational Studies in Epidemiology

## Data Availability

All data generated or analyzed during this study are included in this published paper.
